# The role of the tissue factor and its inhibitor in the development of subclinical atherosclerosis in people living with HIV

**DOI:** 10.1371/journal.pone.0181533

**Published:** 2017-07-27

**Authors:** Katarzyna Barska, Wiesława Kwiatkowska, Brygida Knysz, Katarzyna Arczyńska, Maciej Karczewski, Wojciech Witkiewicz

**Affiliations:** 1 Wrovasc–Integrated Cardiovascular Centre, Regional Specialist Hospital, Research and Development Center in Wroclaw, Wroclaw, Poland; 2 Department of Angiology, Regional Specialist Hospital, Research and Development Center in Wroclaw, Wroclaw, Poland; 3 Department of Infectious Diseases, Wroclaw Medical University, Wroclaw, Poland; University of Pittsburgh Centre for Vaccine Research, UNITED STATES

## Abstract

**Introduction:**

HIV infection is associated with an increased risk of cardiovascular disease in connection with atherosclerosis and thromboembolic complications. The pathogenesis of atherosclerosis is still unclear in this group of patients. Studies on pathogenesis of atherosclerosis in the general population emphasize the role of the extrinsic pathway of blood coagulation, particularly the tissue factor (TF) and tissue factor pathway inhibitor (TFPI). The effect of persistent activation of the immune system on enhanced expression of TF on the surface of monocytes in subjects infected with HIV is known to be correlated with the level of HIV RNA in blood serum.

**Study aim:**

The aim of this study was to evaluate the concentration of TF and its inhibitor TFPI in blood plasma, the impact of traditional and non-traditional cardiovascular risk factors on their concentration and the impact of both markers of haemostasis on the severity of subclinical atherosclerosis as assessed by the intima-media measurement of the carotid artery in HIV infected patients.

**Materials:**

The study included 121 HIV-infected people with known clinical, immunological and virological status. The control group consisted of 42 healthy individuals, selected in terms of age and sex.

**Results and conclusions:**

Higher concentrations of TF occurred in HIV-infected patients with a low current plasma HIV RNA level, nadir CD4+ T-cell count and longer duration of cumulative antiretroviral treatment. In multivariate analysis, it was the length of cumulative NRTI treatment that impacted on the concentration of TF. The determinants of cardiovascular disease (CVD) risk factors and inflammatory markers did not show any effect on the concentrations of TF.

The TFPI level in HIV-infected patients was significantly higher than in the control group and was negatively correlated with the current level of HIV RNA and nadir CD4+ T-cell count, being higher in patients subjected to antiretroviral treatment. It was shown that the higher the cardiovascular risk and the higher the levels of total cholesterol, low-density lipoprotein cholesterol (LDL) and non-high-density lipoprotein cholesterol (non-HDL), the higher the concentrations of TFPI observed. The levels of TF and TFPI were positively correlated with carotid intima media thickness (cIMT); in the multivariate analysis, TF, non-HDL cholesterol and lifetime smoking (pack-years) independently affected the growth of cIMT. A similar effect on cIMT was demonstrated by TFPI.

## Introduction

People infected with HIV demonstrate greater severity of subclinical atherosclerosis and a higher incidence of cardiovascular diseases (CVD). Discussions revolving around the causes of this condition point to the role of the HIV infection per se, the metabolic consequences of antiretroviral (ARV) treatment and the severity of certain traditional risk factors in this population. The effects of chronic inflammation, leading to endothelial cell activation and disturbances in the coagulation system, are also stressed [1].

Stimulation of the immune system by HIV leads to increased expression of the tissue factor (TF) on the surface of monocytes [2]. TF is a most important factor that initiates coagulation cascade via the extrinsic pathway. It is assumed that the thrombogenicity of the atherosclerotic plaque depends on the presence of TF in the plaque. Under physiological conditions, endothelial cells and monocytes do not exhibit TF expression [3]. TF expression is induced by interleukin-1beta (IL-1β), tumor necrosis factor alfa (TNF-α), CD40L, thrombin, oxidized low-density-lipoprotein (oxLDL), angiotensin II, vascular endothelial growth factor (VEGF) and bacterial lipopolysaccharide (LPS) [2–4]. Additionally, the most important risk factors for atherosclerosis, such as arterial hypertension, diabetes and tobacco smoking, increase the concentration of TF in the blood [3]. In late atherosclerotic lesions the presence of TF is also observed on the surface of foam cells, endothelial cells and vascular smooth muscles. TF is also found in the plaque's necrotic core, derived from the apoptotic bodies of foam cells, macrophages and lymphocytes [3,4]. In recent years, studies have demonstrated the important role of circulating or ‘blood borne’ TF in the process of forming a clot in a vessel [3,4].

This form is bound by microparticles [MP] or membrane vesicles, released from platelets, endothelial cells, smooth muscle cells, monocytes and lymphocytes. Because of the presence of P selectin glycoprotein ligand 1 (PSGL-1) on the surface of MP, it was found that they are capable of attaching themselves to the activated platelets where damaged vessels and thrombus formations occur [5–8].

The main factor inhibiting extrinsic coagulation is the tissue factor pathway inhibitor (TFPI). TFPI is primarily synthesized by endothelial cells and blood platelets, monocytes and fibroblasts [4,9]. Most of the circulating TFPI is associated with plasma lipoproteins, mainly low-density-lipoproteins (LDL), and high-density-lipoproteins (HDL) to a lesser degree [10]. The presence of TFPI in atherosclerotic lesions is considered to be the main mechanism limiting the thrombogenicity of the atherosclerotic plaque and preventing the re-occlusion of blood vessels [3,4]. Studies have shown elevated levels of TFPI in patients with peripheral arterial disease of the lower limbs, Buerger's disease, hyperlipidaemia, coronary heart disease and diabetes [3,4].

The increase in TF expression as well as the endothelial dysfunction and secretion of the pro-inflammatory cytokines is associated with increased prothrombotic properties of blood in people living with HIV. It has been observed that the greater the severity of infection (AIDS, CD4+ T-cell count <200/μl), the greater the risk of thromboembolic complications [11–13]. The expression of TF correlates with the level of HIV RNA in blood serum and with the concentration of soluble receptors for LPS—CD14 (sCD14), which are considered markers of progression of HIV infection [14]. In addition, a correlation has been demonstrated between the level of TF and the concentration of D-dimer in the blood [2].

To our knowledge this is the first report concerning information on the TFPI levels in people infected with HIV.

## Study aim

The aim of this study was to evaluate the concentration of the tissue factor and its inhibitor in blood plasma, assess the impact of traditional and non-traditional cardiovascular risk factors on their concentrations and analyze the impact of both markers of haemostasis on the severity of subclinical atherosclerosis as assessed by the intima-media measurement of carotid arteries in patients infected with HIV.

## Materials and methods

### Ethics considerations

Only blood sampling was an invasive procedure. The protocol was evaluated by the ethics committee and the approval of the local Bioethic Committee's was obtained (KB/no/3/2008). Both HIV-infected patients and patients from the control group provided informed consent to participate in the study, according to the Helsinki Declaration.

### Study area

The study was performed in Lower Silesia, Poland.

### Study design and duration, study population

It was an observational, cross-sectional study which consisted of two groups: the study group of HIV- infected patients and control group matched for age and gender, generally fit. Duration of the study was 4 years. Data characterizing these groups, as well as the parameters of subclinical atherosclerosis, cardiovascular risk and risk factors, data on the virological status, immunological status and antiretroviral treatment were part of a larger observation and follow up [13,15].

The study was conducted in two groups:

#### 1. Study group

The consecutive, routinely scheduled HIV-positive patients attending the Acquired Immunodeficiency Syndrome Outpatient Clinic who fulfilled the inclusion and exclusion criteria were invited to participate in the study. One hundred twenty one patients (average age 41.1±9.4, proportion of men 66.1%) were included in the study. The HIV infection characteristics of the patients living in the Lower Silesia region in which our study was conducted are similar (in the course, route of transmission, co-infections, prevention and treatment) and representative of the entire country.

#### 2. Control group

The control group included HIV-negative age- and gender-matched healthy subjects recruited contemporaneously (average age 42.3±11.1, proportion of men: 62%) from the study group region, not meeting the project's clinical and laboratory exclusion criteria. These healthy subjects were recruited at random through two general practices in the region's capital and in the province. Seventy three subjects agreed to participate, of whom 31 were not included—two because of positive HBs antigen, 18 because of data in the medical history indicating the occurence of cardiovascular disease or significant chronic disorders and 11 persons missed the study visit.

### The inclusion and exclusion criteria

The inclusion criteria comprised an identified HIV infection and the respondent's informed consent to participation in the study. The following exclusion criteria for study and control group were: currently recognized acute medical condition, currently recognized AIDS, creatinine levels >2 mg/dL, more than fivefold increase of aminotransferase levels. Additionally the history of cardiovascular diseases was the exclusion criterion for the control group. The HIV-infected patients commenced the tests with a known clinical, immunological and virological status with regard to both HIV infection and hepatitis B (HBV) and/or hepatitis C (HCV) co-infection. The study group included 121 patients and the control group—42 individuals. The characteristics of the study and control groups are shown in Table 1.

**Table 1:** Characteristics of the study group and control group: The parameters of subclinical atherosclerosis, cardiovascular risk and risk factors. Data on the virological status, immunological status and antiretroviral treatment.

Variable	Study group n = 121	Control group n = 42	P-value
Age, (years)*	40 (34–46)	44 (31–51)	0.44
Gender, (No. of men) n (%)	80 (66%)	26 (62%)	0.62
BMI*	23.068 (21.15–25.07)	25.595 (23.89–27.44)	**0.0001**
cIMT, (mm)*	0.655 (0.59–0.79)	0.541 (0.46–0.62)	**0.0001**
Atherosclerotic plaque, n (%)	34 (28.1%)	7 (16.7%)	0.14
Pack years*	14 (4–25)	0 (0–13)	0.39
Cigarette smoking, n (%)	70 (57.9%)	14 (33.3%)	**0.0001**
Hypertension, n (%)	53 (43.8%)	10 (23.8%)	**0.022**
Total cholesterol, (mg/dl)*	192 (157–222)	208 (186–236)	**0.013**
Non-HDL cholesterol, (mg/dl)*	133 (104.5–166)	149 (119–175)	0.069
LDL cholesterol, (mg/dl)*	104 (81–133)	128 (97–151)	**0.006**
Cholesterol HDL, (mg/dl)*	50 (39.75–62.5)	58.5 (47–71)	**0.005**
Triglycerydes, (mg/dl)*	131 (90–188)	100.5 (68–137)	**0.012**
CRP, (mg/l)*	0.68 (0.188–1.51)	0.72 (0.185–1.52)	0.96
Fasting glucose, mg%*	91 (86–96.5)	93.5 (87–101)	0.11
Insulin, (uj/ml)*	7.25 (5.2–10.4)	7 (4.4–10.9)	0.56
HOMA-IR*	1.63 (1.1–2.23)	1.48 (0.980–2.67)	0.91
Fibrinogen, (g/l)*	2.6 (2.3–3.1)	2.9 (2.6–3.3)	**0.013**
Cardiovascular family history, n (%)	39 (32.2%)	5 (11.9%)	**0.011**
Cardiovascular risk NCEP ATP III*	2 (1–6)	2 (0.99–5)	0.38
TNF-α, (pg/ml)*	21.8 (19.625–23.825)	21.85 (18.4–28.4)	0.99
IL-1β, (pg/ml)*	5.8 (5.1–7.9)	6.8 (5.5–9.7)	**0.015**
Route of infection HTX/IDU/MSM	32 (26.4%)/55 (45.5%)/34 (28.1%)	**-**	**-**
Duration of HIV infection, (years)*	8 (4–13.5)	**-**	**-**
History of AIDS, n (%)	33 (27.3%)	**-**	**-**
HCV co-infection, n (%)	66 (54.5%)	**-**	**-**
Past HBV infection, n (%)	28 (23.1%)	**-**	**-**
Current CD4+ T cells (cells/μl) *	503 (397.75–688.5)	**-**	**-**
Nadir CD4+ T cells, (cells/μm)*	213 (74–314)	**-**	**-**
Current HIV RNA, (copies/ml)*	40 (40–50)	**-**	**-**
Undetectable HIV RNA, n (%)	96 (81.4%)	**-**	**-**
Zenith HIV RNA (copies/ml)*	38669 (6 581–182 500)	**-**	**-**
ARV treatment, n (%)	110 (90.9%)	**-**	**-**
Duration of ARV treatment, (years)*	5 (2–9)	**-**	**-**
Cumulative NRTI treatment time, (years)*	8.785 (4.03–13.98)	**-**	**-**
Cumulative NNRTI treatment time, (years)*	0 (0–2)	**-**	**-**
Cumulative PI treatment time, (years)*	3.79 (0.71–7.37)	**-**	**-**
Cumulative ARV treatment time (years)*	16.61 (8.13–24.97)	**-**	**-**

Notes: HTX, heterosexual; IDU, intravenous drug user; MSM, men who have sex with men

*—median (IQR), n (%)–absolute number (percent), remaining data—arythmetic mean

The study protocol included: medical history, the occurrences of cardiovascular diseases and risk factors, physical examination, resting ECG and anthropometric measurements with a standard calculation of the body mass index (BMI). A positive family history of cardiovascular diseases was based on the National Cholesterol Education Program Adult Treatment Panel III (NCEP ATP III) criteria [16]. Additionally, the so-called lifetime smoking (pack-years) and cardiovascular risk were worked out according to the NCEP ATP III calculator.

An assessment was made regarding HIV infection data that included the following: infection duration, route of infection, history of AIDS, HCV and HBV co-infections, current CD4 cell count, nadir CD4, HIV RNA and zenith HIV RNA, type of ARV used during the study period, duration of ARV treatment (in years), cumulative treatment time of the antiretroviral treatment and for the individual classes of antiretroviral drugs.

In assessing the impact of antiretroviral therapy on concentration of tested haemostasis markers, only ARV-treated patients were taken into account. 110 individuals received cART (combined antiretroviral therapy) in combination of nucleoside reverse transcriptase inhibitors (NRTI) and protease inhibitors (PI) or non-nucleoside reverse transcriptase inhibitors (NNRTI). The treatment was previously changed several times, mainly because of the toxicity of drugs, and therefore could not be subjected to statistical analysis of the impact of individual antiretroviral drugs on the level of TF and TFPI. The other respondents have not been treated during the observation.

### Echo-Doppler examination

In all subjects an echo-Doppler examination of the carotid arteries was performed, registering ultrasound images for further treatment, in order to assess subclinical atherosclerosis with a computer-measured carotid intima-media thickness (cIMT). The carotid ultrasound was performed in B-mode with a high-resolution USG LOGIQ 7 machine from GE with a broadband linear probe of 6–12 MHz and 5x magnification. Four series of images were collected for each patient: common carotid arteries and the bulb on both sides. The images were recorded in three projections for each series: anterior, lateral and posterior. Far wall images of the distal common carotid arteries and bulbs were recorded parallel to the probe surface at the smallest artery diameter and then saved on the hard drive and on DVDs. A place with local cIMT thickening exceeding 1.5 mm was adopted as atherosclerotic plaque [17].

### Laboratory tests

After 14 hours of fasting, blood was collected for laboratory testing, performed by standard laboratory methods: haematology test, levels of aminotransferases, creatinine, glucose, total cholesterol (TC), HDL fraction, triglycerides (TG), fibrinogen, D-dimers, wide range C-reactive protein (CRP) and insulin. Concentration of LDL cholesterol was calculated according to the Friedewald rule, non-HDL cholesterol according to the TC—HDL formula and insulin resistance with the HOMA-IR indicator (homeostasis model of assessment: insulin resistance) according to the following formula: fasting insulin levels (uIU/ml) x fasting glucose (mmol/l) /22.5. Insulin resistance was diagnosed with a HOMA-IR value > 2.5

Blood collected for testing of TF and TFPI, and also pro-inflammatory markers concentration levels was put into S-Monovette test tubes containing sodium citrate. These were centrifuged for 15 minutes at +4°C at 2000 x g. Eppendorf micro test tubes were filled with 200 μl of plasma. The prepared samples were banked at -70°C. TF concentration was determined by enzyme immunoassay (ELISA) of the citrate plasma with a reagent kit (IMUBIND® Tissue Factor ELISA from American Diagnostica) and expressed as pg/ml; the test detected TF, TF-apo and TF/VII complex. The TFPI enzyme immunoassay (ELISA) in the citrate plasma was performed with a reagent kit (IMUBIND® Total TFPI ELISA from American Diagnostica) and expressed as ng/ml; the test detected the full-length TFPI and ‘truncated’ TFPI forms that remained bonded with TF or factor VII.

The TNF-α enzyme immunoassay (ELISA) in the plasma was performed with a reagent kit (Human TNFalfa ELISA kit from Diaclone) and expressed as pg/ml. The IL-1β enzyme immunoassay (ELISA) in the plasma was performed with a reagent kit (Human IL-1β ELISA kit from Diaclone) and expressed as pg/ml.

Anti-HIV, anti-HBc and anti-HCV antibodies were examined in the control group using the enzyme immunoassay method with microparticles. Plasma HIV RNA levels were tested by the use of Roche Amplicor 1.0 standard assay and CD4 T cell counts by FACScan (Becton-Dickinson) flow cytometry method.

### Statistical analyses

The normality of data was tested with the Pearson D' Agostino test. Because normal distribution of variables did not apply, analyses were performed with non-parametric tests. The results of quantitative variables were presented as medians and quartile ranges or in the case of qualitative characteristics with numbers and percentages. The analysis of variations between two quantitative characteristics was performed with the Mann-Whitney test. The analysis of the relationship between two qualitative characteristics was tested with the χ2 test (chi square) or Fisher's exact test in the case of a small number of subgroups. Linear regression analysis was performed to investigate the effect of variables on the characteristics of atherosclerosis. Multivariate regression analysis was performed to assess the independent impact of variables on cIMT and multivariate logistic regression analysis to assess the risk of plaque. The level of significance was set at p<0.05. Program R for Windows (version 3.1.2) was used for the analyses.

## Results

### CVD risk factors and concentrations of TF and TFPI in blood plasma of study group and control group

HIV-infected patients had significantly higher levels of TFPI (p = 0.0001) than the control group; however, differences in the concentration of TF between the two groups were not demonstrated (Table 2). The TF levels were significantly higher in patients on ARV in comparison with HIV positive patients without ARV treatment, however we did not observe significant differences with control group. The levels of TFPI were significantly higher in patients on ARV comparing with these not treated and control group.

**Table 2:** Comparisons of plasma levels of TF and TFPI between subjects from the study and control group (Mann-Whitney test).

Parameter	Study group (HIV positive subjects) n = 121	Control group n = 42	p-value
**TF (pg/ml)**	245 (160.5–353.15)	218 (160–292)	0.35
**TFPI (ng/ml)**	58.6 (47–70.075)	49 (42.1–55.7)	**0.0001**
	**Study group: on ARV** n = 110	**Control group** n = 42	
**TF (pg/ml)**	257,3 (165,5–374)	218,35 (160–292)	0,18
**TFPI (ng/ml)**	59,65 (51,3–71,3)	49 (42,1–55,7)	**<0,0001**
	**Study group: on ARV** n = 110	**Study group: ARV naïve subjects** n = 11	
**TF (pg/ml)**	257.3 (165.5–374)	194.3 (132–227.3)	**0.037**
**TFPI (ng/ml)**	59.7 (51,3–71.3)	45 (40.5–50.0)	**0.0003**

Note: variable are expressed as median (IQR)

A significant positive, weak correlation was observed between the concentrations of TF and TFPI in the HIV-positive cohort (p<0.001, r = 0.389), whereas weak inverse correlation was observed in the control group (p = 0.034, r = -0.328) (S1 Fig).

Among the traditional cardiovascular risk factors high concentrations of total cholesterol, non-HDL and LDL cholesterol significantly increased the concentration of TFPI. A positive correlation has been observed between TFPI level and the cardiovascular risk according to NCEP-ATP III criteria (Table 3).

**Table 3:** Effect of cardiovascular disease risk factors on the concentration of TF and TFPI (univariate linear regression).

Parameter	TF			TFPI		
	B	SE	P-value	B	SE	P-value
Age	3.494	1.912	0.07	0.28	0.171	0.277
Gender	-11.76	36.740	0.75	1.606	3.273	0.625
BMI	-0.182	4.953	0.971	-0.444	0.44	0.314
Pack-years	0.094	1.122	0.933	0.075	0.099	0.453
Total cholesterol	-0.123	0.393	0.755	0.106	0.034	**0.002**
Non-HDL cholesterol	-0.363	0.424	0.394	0.089	0.037	**0.018**
LDL cholesterol	-0.139	0.477	0.772	0.113	0.042	**0.008**
HDL cholesterol	0.702	0.759	0.357	0.112	0.067	0.098
Triglycerydes	-0.163	0.208	0.434	0.006	0.019	0.743
CRP	-0.92	2.593	0.724	0.287	0.23	0.215
HOMA-IR	-1.996	11.589	0.864	-0.498	0.964	0.607
Fibrinogen	-5.461	21.775	0.802	1.517	1.936	0.435
D–dimers	0.076	0.040	0.129	0.004	0.004	0.344
Cardiovascular risk NCEP ATP III	0.578	3.286	0.861	0.575	0.288	**0.048**
TNF-ɑ	-0.596	1.949	0.760	-0.225	0.181	0.215
IL-1ß	-0.538	1.661	0.747	0.098	0.157	0.532
HCV co-infection	-15.63	35.007	0.656	-5.411	3.082	0.082
Current Lymph. T CD4+	0.074	0.069	0.285	-0.0006	0.006	0.921
Lymph. T CD4+ nadir	-0.234	0.112	**0.038**	-0.038	0.009	**0.0001**
Current HIV RNA	-0.006	0.003	**0.036**	-0.0005	0.000	**0.023**
Zenith HIV RNA	3.606E-05	0.0001	0.539	7.196E-06	5.179E-06	0.167
Cumulative NRTI treatment time	6.784	2.21	**0.003**	0.148	0.204	0.470
Cumulative NNRTI treatment time	8.682	5.878	0.142	0.498	0.527	0.347
Cumulative PI treatment time	11.545	4.188	**0.007**	0.161	0.385	0.677
Cumulative ARV treatment time	5.52	1.518	**0.0004**	0.134	0.142	0.347

In the examined group, analyses were also performed with univariate linear regression to study the effect of the status of virological and immunological parameters as well as the correlation with antiretroviral treatment time on the concentration of TF and TFPI. The negative effect was demonstrated of current HIV RNA and nadir CD4+ T-cell count on concentration of TF and TFPI. Strong independent effect of cumulative ARV treatment time and NRTI and PI treatment time was recorded on higher concentration of TF (Table 3).

**Fig 1:**
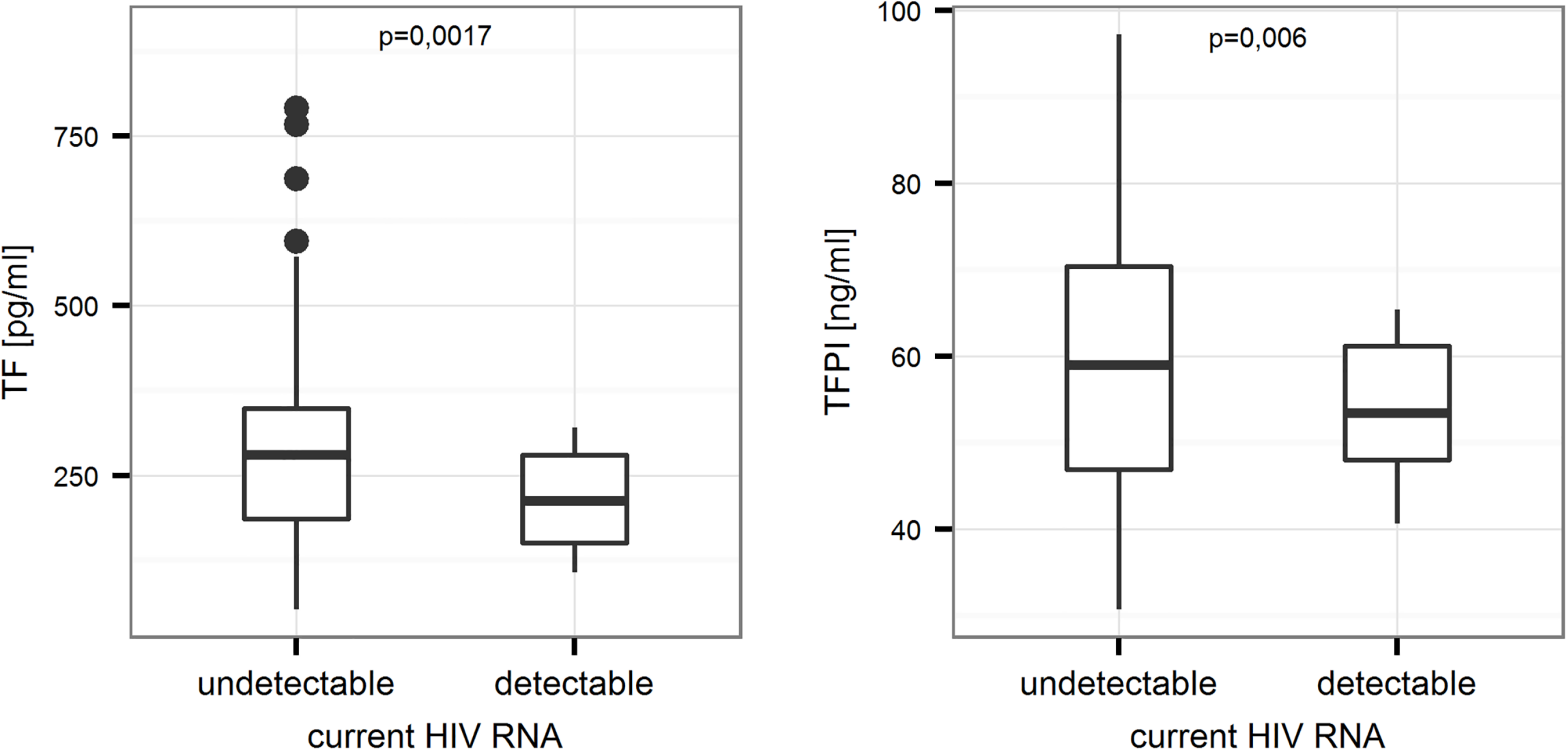
Comparison of concentrations of TF and TFPI between patients with detectable and undetectable levels of HIV RNA in the study group (p = 0.0017 and p = 0.006, respectively). Mann-Whitney test.

Concentrations of TF and TFPI were higher in patients with undetectable HIV RNA (Fig 1).

In the stepwise multivariate regression model, the effect of the cumulative NRTI treatment time on the concentration of TF was found (Table 4).

**Table 4:** Effect of traditional CVD risk factors, inflammatory markers (model 1), virological and immunological parameters, including cumulative antiretroviral PI and NRTI treatment (model 2), on the concentrations of TF (multivariate regression).

Variables	B	SE	P-value
**Model 1**			
Age	4.849	2.58	0.064
Gender	-29.57	49.23	0.55
Hypertension	1.746	48.614	0.971
Pack-years	-0.664	1.509	0.661
Family history (CVD)	-41.077	46.391	0.378
HOMA-IR	-5.335	12.616	0.673
BMI	3.909	6.936	0.575
Non-HDL cholesterol	-0.639	0.533	0.234
TNF-α	-1.217	3.472	0.727
IL-1β	4.938	6.852	0.473
Fibrinogen	-13.808	26.460	0.603
**Model 2**			
Past HBV infection	-72.71	47.27	0.13
HCV co-infection	5.82	38.74	0.88
Current Lymph. T CD4+	0.03	0.09	0.74
Lymph. T CD4+ nadir	-0.15	0.16	0.33
Current HIV RNA	-0.003	0.003	0.36
Zenith HIV RNA	-0.00003	0.00006	0.65
Cumulative NRTI	4.06	2.96	0.17
Cumulative PI	6.86	5.26	0.19
**Stepwise**			
Cumulative NRTI	7.22	2.31	**0.002**

After application of the same multivariate regression model for TFPI we found no effect of traditional CVD risk factors on its concentration. A negative, independent effect was demonstrated of the nadir CD4+ T-cell count on concentrations of TFPI (b = -0.04, SE = 0.01, p = 0.0003).

### Effect of TF and CVD risk factors on the development of subclinical atherosclerosis

**Fig 2:**
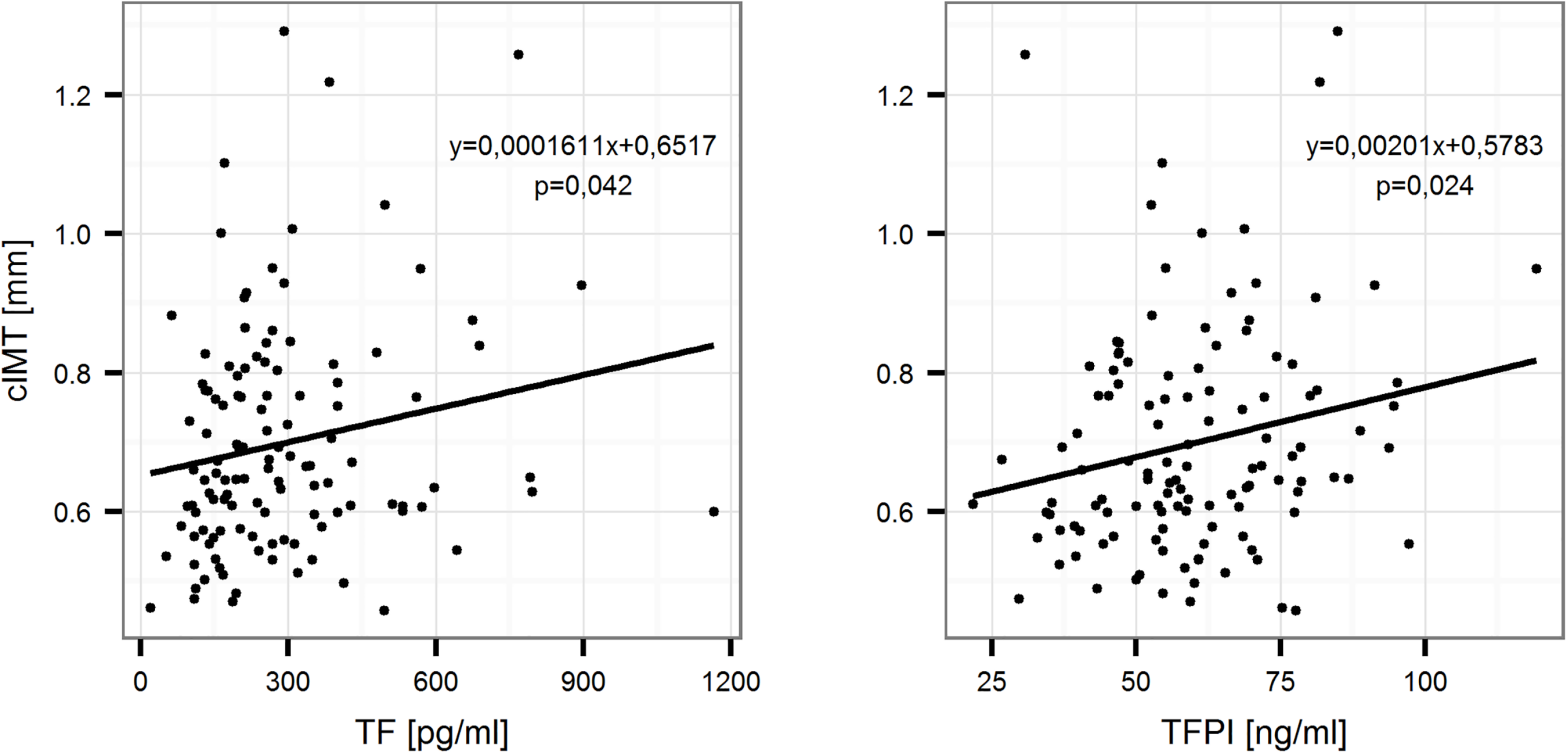
Linear relationship between the concentrations of TF and TFPI and the value of cIMT for the entire study group. Univariate linear regression.

Univariate linear regression analysis revealed a positive correlation between both TF and TFPI with cIMT in the study group (Fig 2).

In the multivariate regression model involving risk factors after removal of variables with the backward elimination method, a positive, independent effect of TF, non-HDL cholesterol levels and value of pack-years on the value of cIMT was demonstrated; however, no effect of TF was shown on the presence of atherosclerotic plaque (Table 5).

**Table 5:** Analysis of the effect of TF and TFPI and risk factors on cIMT (multivariate regression model).

Independent Variables	cIMT (model with TF)			cIMT (model with TFPI)		
	Coeff.	SE	P-value	Coeff.	SE	P-value
(Constant)	0.303			0.260		
TF/TFPI	0.0002	0.0001	**0.014**	0.002	0.0009	0.052
Gender	0.008	0.034	0.816	0.0009	0.034	0.979
Family history CVD	0.006	0.033	0.846	0.004	0.033	0.900
Non-HDL cholesterol	0.001	0.0004	**0.002**	0.0009	0.0004	**0.019**
Pack-years	0.004	0.001	**0.0002**	0.004	0.001	**0.0001**
HOMA-IR	0.003	0.009	0.758	0.003	0.009	0.731
Systolic blood pressure	0.0008	0.0008	0.331	0.001	0.0008	0.229
**Stepwise**						
(Constant)	0.406			0.385		
TF/TFPI	0.0002	0.0001	**0.009**	0.0019	0.0009	**0.045**
Non-HDL cholesterol	0.0012	0.0004	**0.002**	0.0009	0.0004	**0.015**
Pack-years	0.004	0.0009	**0.0001**	0.004	0.0010	**0.0001**

In the same regression model only the independent effect of the current intensity of cigarette smoking on the presence of atherosclerotic plaque was demonstrated (pack-years, p = 0.026).

**Fig 3:**
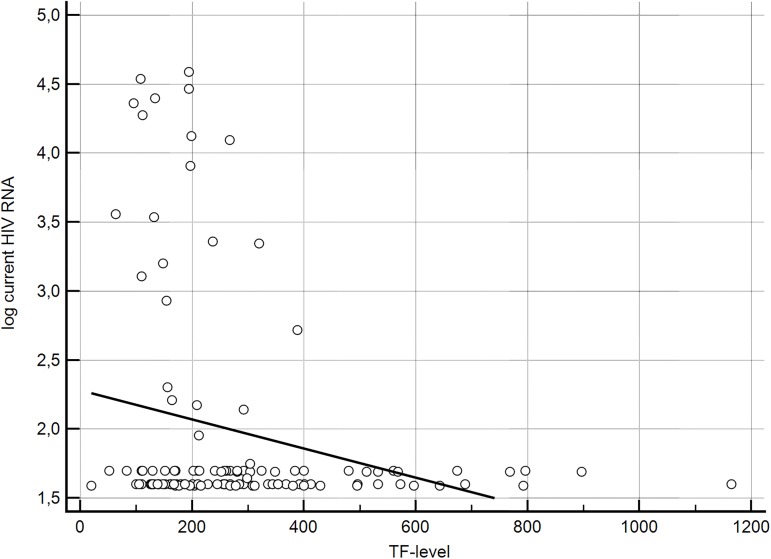
Graphic presentation of the dependency of TF level and HIV RNA level.

Based on the observation, that the concentration of TF correlates with the progression of subclinical atherosclerosis, an additional analysis was performed in order to examine whether the high level of TF were accompanied by such risk factors as HIV RNA plasma level, CD4+Tcell count and traditional risk factors for cardiovascular diseases. We found that TF level > 450 pg/ml was presented only in patients on ARV with HIV RNA level below detection limit. This homogenous group was compared with the group of patients with TF level <450pg/ml. The cut-off point was established on the base of the graphic presentation of correlation between TF level and current HIV RNA level (Fig 3).

Patients with TF> 450 pg/ml were demonstrating undetectable viral load, the significantly higher current CD4+ T-cell count (p = 0.031) and longer duration of HIV infection (p = 0.59). Moreover, patients with a longer cumulative antiretroviral treatment time (p = 0.003), as well as the cumulative PI (p = 0.006) and NRTI treatment time (p = 0.008), had significantly higher levels of TF (S1 Table). Among those with high values of TF there were no respondents who had not been treated. For the purpose of the analysis, in order to increase the strength of the statistical analysis, we combined study group with the control group (163 individuals). Patients with high concentration of TF (>450 pg/ml) were older (44 *vs*. 40 years, p = 0.02), had significantly higher systolic blood pressure (130 mmHg *vs*. 120 mmHg, p = 0.006), arterial hypertension occurred more frequently (66.7% *vs*. 35.4%, p = 0.035) and they also had higher cardiovascular risk (4.5 *vs*. 2, p = 0.059). In addition, they were characterized by greater value of cIMT as compared to patients with low concentration of TF (resp. 0.62 mm *vs*. 0.7, p = 0.036).

## Discussion

In the study group a negative correlation between the concentration of TF, current level of HIV RNA and nadir CD4+ T-cell count was demonstrated. It was proven that the cumulative PI and NRTI treatment time, exhibits a strong, independent effect on the concentration of TF in plasma. In addition, it was observed that patients receiving cART with HIV RNA values below the detection limit, had significantly higher levels of TF. High values of TF were observed only in ARV treated patients. Moreover, patients with high levels of TF were older and had more advanced subclinical atherosclerosis and higher systolic blood pressure values, and were treated longer with the antiretroviral therapy.

In the present study no effect of traditional cardiovascular risk factors on the concentrations of TF was demonstrated.

HIV infection leads to the activation of coagulation processes, which may lead in turn to the development of atherosclerosis and venous thromboembolism. It was observed that the greater the severity of infection (AIDS, CD4+ T-cell count <200 cells/μl), the greater the risk of thromboembolic complications [11,12]. The initiating factor in the coagulation cascade via the extrinsic pathway is the tissue factor. The production of TF in endothelial cells is induced by the pro-inflammatory cytokines (TNF-α and IL-1β) as well as oxLDL, thrombin and VEGF [11,12,14,18]. The concentration of TF in blood serum does not always correlate with TF activity, which is associated with concomitant production of TFPI by the endothelial cells [3,4]. During the course of HIV-1 infection there is an increase in TF expression on the surface of activated monocytes. Funderburg and colleagues demonstrated that the expression of TF on monocytes correlated with the level of HIV RNA, the co-receptor CD14 and the concentration of D-dimers [2,14]. Ford and colleagues also noted the increased expression of TF on the monocytes of people infected with HIV, which was associated with an increased risk of cardiovascular disease [19].

The concentration of TF was higher in the study group than in the control group, but the difference was not statistically significant, despite the increased severity of subclinical atherosclerosis in HIV infected patients. Higher concentrations of TFPI were also found in the HIV-positive cohort than in healthy subjects, possibly because of the short duration of the activity and rapid elimination of TF from the circulation and, at the same time, a high concentration of TFPI, which plays an important role in the removal of secreted TF [4,8,9].

Our observations suggest that in patients receiving cART, who have good response to treatment, with HIV RNA values below detection limit, endothelial injury is mainly the result of the toxicity of PIs and NRTIs, and is manifested by increased levels of TF in the subjects' plasma. Endothelial dysfunction and high values of TF are associated with increased cardiovascular risk, greater severity of subclinical atherosclerotic lesions and hypertension.

We also demonstrated that at low nadir CD4+ T-cell count the concentration of TF were significantly increased. Nadir CD4+ T-cell count reflects a profound immune deficiency in the past, and is also associated with chronic immune activation and persistent microbial translocation [20]. An increasing number of studies show that a low nadir CD4+ T-cell count (<50 cells/μl), is an unfavourable prognostic marker of diseases unrelated to AIDS. Manner and colleagues showed that nadir of CD4+ T-lymphocytes is an independent risk factor for the development of hypertension in people living with HIV [21]. On the other hand, Hsue and colleagues showed that nadir CD4+ cell count <200 cells/μl is an independent predictor of the progression of subclinical atherosclerosis [20]. The available literature did not reveal the described correlation between the concentration of TF and the nadir CD4+ T-cell count. The observed association may be due to continuing, persistent activation of the vascular endothelium.

Significantly higher concentrations of TFPI were revealed in HIV infected people than in the control group. The concentration of TFPI was negatively correlated with the current level of HIV RNA and nadir CD4+ T-cell count. Moreover, it was increased in patients treated with antiretroviral drugs. There was no effect, however, of the cumulative antiretroviral treatment time or the cumulative treatment time with individual classes of drugs on the concentration of TFPI. However, it was dependent on the traditional cardiovascular risk factors: the greater the cardiovascular risk evaluated according to NCEP ATP III criteria, and the higher the concentrations of total cholesterol in plasma, LDL and non-HDL cholesterol, the higher the concentrations of TFPI in our observations.

The TFPI is a primary factor inhibiting the extrinsic blood coagulation system and it participates in the regulation of haemostatic mechanisms [4,8].

Almost 90% of circulating TFPI is associated with plasma lipoproteins [4,10,22,23]. Hansen and colleagues observed a correlation between the TFPI level and the concentration of LDL cholesterol fraction in patients with familial hypercholesterolaemia [22]. Studies by Saito and colleagues, however, revealed significantly higher concentrations of TF and TFPI among people with hyperlipidaemia who showed no signs of cardiovascular disease [23]. Moreover, Sakkinen et al. described a correlation between TFPI and the concentration of fibrinogen and LDL levels in a cohort of elderly people. They also pointed to the relationship of TFPI with subclinical atherosclerotic lesions, suggesting that increased levels of TFPI in the serum may reflect the degree of endothelial injury [24].

In the present study a correlation between the concentration of TFPI and particular atherogenic cholesterol fractions was reported, but no relationships were shown with the fibrinogen level. A negative correlation was observed between TFPI and the level of HIV RNA.

High concentrations of TFPI among individuals with undetectable HIV RNA may, as in the case of TF, be indicative of endothelial injury resulting from antiretroviral treatment. As a matter of fact, we did not notice the cumulative effect of antiretroviral therapy or cumulative treatment using a particular class of drugs on the value of TFPI; however, the TFPI concentration was significantly higher among individuals who were treated (p = 0.0003) than among those who had not undergone antiretroviral therapy.

The present study also revealed a weak positive correlation between the concentrations of TF and TFPI in the study group. This may be because of the increased severity of subclinical atherosclerosis in HIV-infected individuals, whereas the increased TFPI concentration in serum is a response to increased expression of TF by injured endothelial cells and activated monocytes. Falciani and colleagues described a positive relationship among people with coronary heart disease in general population [25]. This may be because of the increased severity of subclinical atherosclerosis in HIV-infected individuals, whereas the increased TFPI concentration in serum is a response to increased expression of TF by injured endothelial cells and activated monocytes.

The results of studies in recent years have indicated the involvement of extrinsic coagulation pathways in haemostatic disorders and thromboembolic complications in the course of cardiovascular diseases and atherosclerosis [8,26,27]. The main role is played by the tissue factor, which is the most important factor initiating the coagulation cascade via the extrinsic pathway [3,4,8]. It is assumed that thrombogenicity of the atherosclerotic plaque depends on the presence of tissue factor in it [3,25].

TF and TFPI significantly influenced the value of cIMT in the cohort. In the multivariate regression model an independent effect of TF, TFPI, non-HDL cholesterol and lifetime smoking on the value of cIMT was demonstrated.

The increase in TF expression, the endothelial dysfunction and the secretion of proinflammatory cytokines are associated with increased prothrombotic properties of blood in people living with HIV [11,14,18,26]. In a retrospective study by Ford and colleagues higher concentrations of soluble TF were observed in HIV-infected patients who had suffered from cardiovascular event [19]. In the available literature there are no reports on the effects of TF on the development of subclinical atherosclerosis in HIV-infected patients. In studies of the general population a relationship has been demonstrated between the monocyte TF activity and the value of cIMT in the metabolic syndrome or in patients with arterial hypertension [27,28].

The presence of TFPI in atherosclerotic lesions is considered as the main mechanism limiting the thrombogenicity of the atherosclerotic plaque; it also prevents vessel reocclusion. TFPI released from endothelial cells and circulating in the blood plays an important role in inhibiting TF, limiting its procoagulant activity [4,25]. The role of TFPI in development of atherosclerotic lesions in the course of HIV infection is unknown.

This is the first study demonstrating the effect of TF and TFPI on the severity of subclinical atherosclerosis in patients living with HIV and indicating the relationship of their concentrations with antiretroviral treatment.

There were some limitations of this study. First, our study and control groups were relatively small and we could not achieve adequate power of our statistical analysis. There was also no possibility for comparison of our results with another contemporary analysis in the Polish general population. Second, due to frequent changes in the treatment, could not be subjected to statistical analysis the impact of individual antiretroviral drugs on the level of TF and TFPI. In addition, tested states of plasma TF (TF, TF-apo and TF/VII complex) could not detect the TF activity. It is believed that the functionally active form of TF is microparticle TF, which represents only small portion of circulating TF [5,6].

## Conclusions

Nucleoside reverse transcriptase inhibitors had impact on TF plasma level and may contribute to a systemic hypercoagulable state. Findings of our study identify TF and TFPI as potential markers for advanced subclinical atherosclerosis in HIV-infected patients and could have practical applications in primary prevention of cardiovascular diseases in people living with HIV.

## Supporting information

S1 Fig Correlation between TF and TFPI in study group (SG) and control group (CG). (TIF)

S1 Table Study group, comparison of subgroups with TF<450pg/ml and >450pg/ml. (PDF)
