# Supplementary figures and images for: The role of the tissue factor and its inhibitor in the development of subclinical atherosclerosis in people living with HIV
Source: PLoS One. 2017 Jul 27;12(7):e0181533. doi: 10.1371/journal.pone.0181533 (PMC5531520; doi:10.1371/journal.pone.0181533)

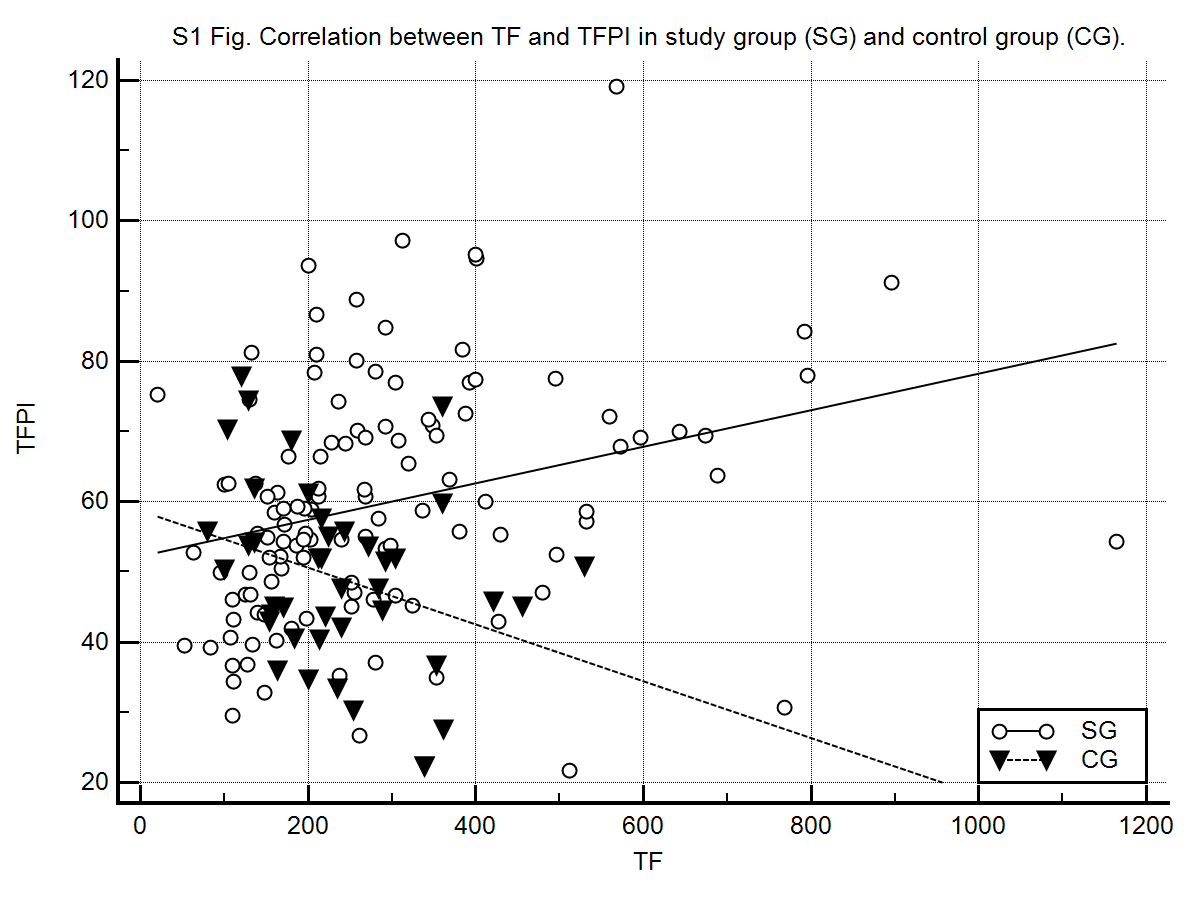

Supplement: S1 Fig — (TIF) [file pone.0181533.s001.tif]
