# Supplementary material for: The role of the tissue factor and its inhibitor in the development of subclinical atherosclerosis in people living with HIV
Source: PLoS One. 2017 Jul 27;12(7):e0181533. doi: 10.1371/journal.pone.0181533 (PMC5531520; doi:10.1371/journal.pone.0181533)
Supplement: S1 Table — (PDF) [file pone.0181533.s002.pdf]

S1 Table. Study group, characteristic of subgroups with TF<450pg/ml and >450pg/ml

Parameter	TF		p-value
	< 450 pg/ml	>450 pg/ml	
**Route of infection HTX/IDU/MSM (%)**	24.8 / 47.5 / 27.7	38.9 /27.8 / 33.3	0.1
**Duration of HIV infection, (years)***	8 (3 -.7 23)	11 (8 - 18)	0.059
**AIDS, n (%)**	30 (29.7%)	3 (16%)	0.26
**HCV co-infection, n (%)**	25 (24.75%)	1 (5.6%)	0.069
**Past HBV infection, n (%)**	56 (55.45%)	8 (44.45%)	0.39
**Current CD4+ T cells (cells/µl) ***	499 (384 - 648)	673 (477 - 794)	**0.031**
**Nadir CD4+ T cells, (cells/µm)***	233 (93 - 349)	137 (46 - 274)	0.086
**Current HIV RNA, (copies/ml)***	40 (40 - 50)	44.5 (40 - 49)	0.29
**Undetectable HIV RNA, n (%)**	22 (22.5%)	18 (100%)	**0.026**
**Zenith HIV RNA (copies/ml)***	32150 (6581– 149720)	64300 (35825 - 226130)	**0.07**
**ARV treatment, n (%)**	88 (87.12%)	18 (100%)	0.11
**Duration of ARV treatment, (years)***	4 (1.6 - 8)	9 (6 - 11)	**0.002**
**Cumulative NRTI treatment time (years)***	6.44 (2.173 -- 11.667)	13.13 (5.73 -- 17.18)	**0.008**
**Cumulative NNRTI treatment time (years)***	0 (0 - 1.35)	0 (0 - 2.42)	0.76
**Cumulative PI treatment time (years)***	2.25 (0 - 5.822)	7.115 (3.91 - 10.06)	**0.006**
**Cumulative ARV treatment time (years)***	11.71 (3.98 - 20.965)	21.305 (14.68 - 27.14)	**0.003**
